# Supplementary material for: Multimodal sensor fusion in the latent representation space
Source: Sci Rep. 2023 Feb 3;13:2005. doi: 10.1038/s41598-022-24754-w (PMC9898225; doi:10.1038/s41598-022-24754-w)
Supplement: Supplementary file 1 — Supplementary Information. [file 41598_2022_24754_MOESM1_ESM.pdf]

Supplementary Information

S1 Approximations to variational posterior

Given the objective, the variational joint posterior $q_\phi(z|x_{1:M})$ can be learned by training one single encoder network that takes all modalities $X_{1:M}$ as input to explicitly parametrize the joint posterior. This is our baseline model and an example for $M = 2$ modalities is given in Fig. S1. However, this approach requires all modalities to be present at all times, thus making cross-modal generation difficult. Alternatively, the joint variational posterior can be modelled using the following approaches:

Variational Product of Experts

In this section we reproduce the arguments from[1]. The first option is to approximate the joint variational posterior as a product:

$$q_\phi(z|x_{1:M}) \equiv p(z)\prod_{m=1}^{M} q_{\phi_m}(z|x_m). \tag{S1}$$

In case of a missing expert, we assume: $q_{\phi_m}(z|x_m) = 1$.

For a system of N modalities, 2^N inference networks need to be specified, $q(z|X)$ for each subset of modalities $X \subseteq \{X_1, X_2, \ldots, X_M\}$. The optimal inference network $q(z|x_1, \ldots, x_N)$ would be the true posterior $p(z|x_1, \ldots, x_N)$. The conditional independence assumptions in the generative model imply a relation among joint- and single-modality posteriors[1]:

$$\begin{aligned}
p(z|x_1,\ldots,x_N) &= \frac{p(x_1,\ldots,x_N|z)p(z)}{p(x_1,\ldots,x_N)} \\
&= \frac{p(z)}{p(x_1,\ldots,x_N)}\prod_{i=1}^{N} p(x_i|z) \\
&= \frac{p(z)}{p(x_1,\ldots,x_N)}\prod_{i=1}^{N} \frac{p(z|x_i)p(x_i)}{p(z)} \\
&= \frac{\prod_{i=1}^{N} p(z|x_i)}{\prod_{i=1}^{N-1} p(z)} \cdot \frac{\prod_{i=1}^{N} p(x_i)}{p(x_1,\ldots,x_N)} \\
&\propto \frac{\prod_{i=1}^{N} p(z|x_i)}{\prod_{i=1}^{N-1} p(z)}.
\end{aligned} \tag{S2}$$

If we approximate $p(z|x_i)$ with $q(z|x_i) \equiv \tilde{q}(z|x_i)p(z)$, where $\tilde{q}(z|x_i)$ is the underlying inference network, the quotient term can be omitted[1]:

$$\begin{aligned}
p(z|x_1,\ldots,x_N) &\propto \frac{\prod_{i=1}^{N} p(z|x_i)}{\prod_{i=1}^{N-1} p(z)} \\
&\approx \frac{\prod_{i=1}^{N} [\tilde{q}(z|x_i)p(z)]}{\prod_{i=1}^{N-1} p(z)} \\
&= p(z)\prod_{i=1}^{N} \tilde{q}(z|x_i).
\end{aligned} \tag{S3}$$

Equation (S3) implies that we can use a Product-of-Experts (PoE), including a "prior expert" (e.g., spherical Gaussian), as the approximating distribution for the joint-posterior. This derivation is easily extended to any subset of modalities yielding $q(z|X) \propto p(z)\prod_{x_i \in X} \tilde{q}(z|x_i)$.

Variational Mixture of Experts

$$q_\phi(z|x_{1:M}) \equiv \sum_{m=1}^{M} \frac{1}{M} q_{\phi_m}(z|x_m), \tag{S4}$$

where the above assumes an equitable distribution of power among experts. Non-uniform weights can also be used. Missing expert: $q_{\phi_m}(z|x_m) = 0$.

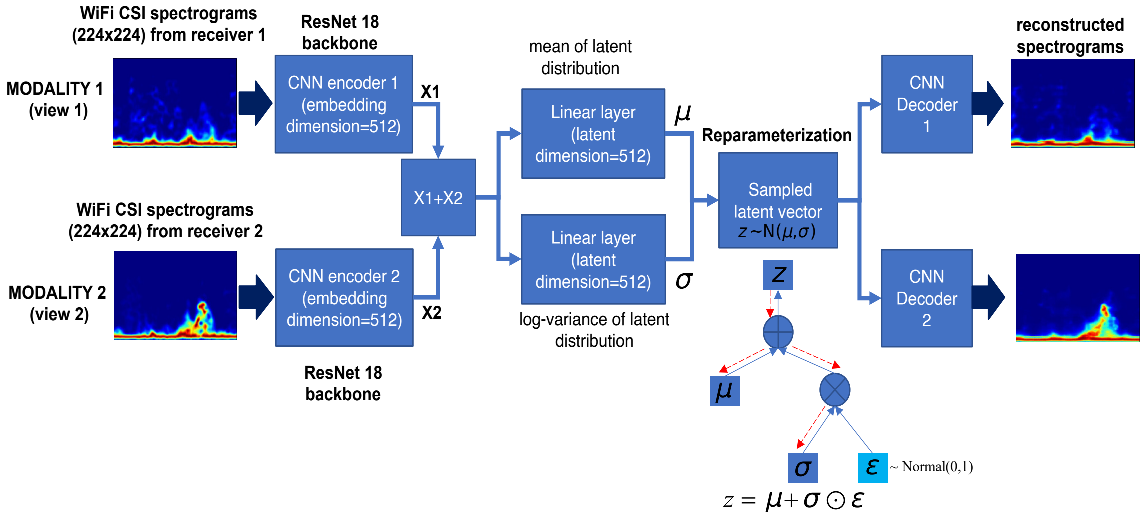

Figure S1. M-VAE for a full data case: Single encoder network takes all modalities.

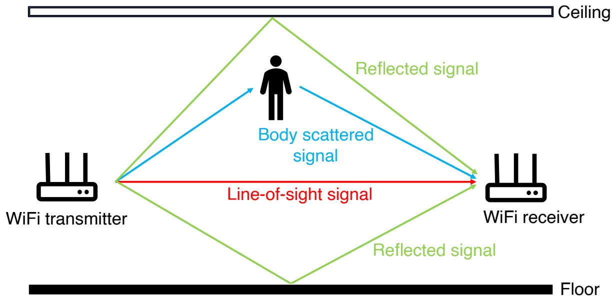

Figure S2. Opportunistic Passive WiFi Radar.

S2 Signal processing pipelines for passive WiFi radar

A typical scenario for opportunistic passive WiFi Radar is depicted in Figure S2. This is an extremely challenging problem since the WiFi waveform was not specifically designed to lend itself to Radio-Frequency (RF) imaging. In addition, commercial WiFi chipsets have noisy RF chains and tend to suffer from phase drifts. The WiFi backscatter does contain information about the dynamic changes in the radio channel which is incapsulated in the Channel State Information (CSI). Dedicated tools need to be used to extract the CSI from WiFi network interface cards such as Atheros[2] or Intel 5300 (IWL5300)[3]. The raw CSI data is obtained as a 3-dimensional (3D) matrix per transmitted packet, with $n_t \times n_r \times N_{\mathrm{sc}}$ complex values, where n_t is the number of transmit antennas, n_r is the number of receive antennas and N_{sc} is the number of subcarriers. Since the raw CSI data is very noisy in nature, the Discrete Wavelet Transform (DWT) technique can be used to filter out in-band noise and preserve the high frequency components, thus avoiding the distortion of the signal[4]. Afterwards, median filtering can be used to remove any undesired transients in the CSI measurements which are not due to human motion. The Intel 5300 chipset has a 3×3 antenna configuration and only 30 subcarriers are reported by this chipset. Thus the number of complex values per packet is equal to $3 \times 3 \times 30 = 270$. Considering a packet rate as high as 1.6 kHz, this results in a significant amount of data that needs to be processed. Therefore, we also apply Principal Component Analysis (PCA) to reduce the computational complexity of such high dimensional data. PCA identifies the time-varying correlations between the CSI streams which are optimally combined to extract only a few components that represent the variations caused by human activities. Finally, we convert the resultant data into spectrograms (time-frequency domain) using Short Time Fourier Transform (STFT), which are similar to those generated by Doppler radars. The CSI is highly sensitive to the surrounding environment and signal reflections from the human body result in different frequencies when performing different activities. The Doppler spectrogram generated from STFT helps to identify the change of frequencies over time. The generated spectrograms can be directly fed to CNNs to automatically identify a set of features, which can ultimately be used in downstream tasks. The CSI system consisted of two receivers. For more details on the experimental setup of the data collection, the interested reader is kindly referred to[5]. Each receiver can be seen as another view of the human activity being performed in the environment.

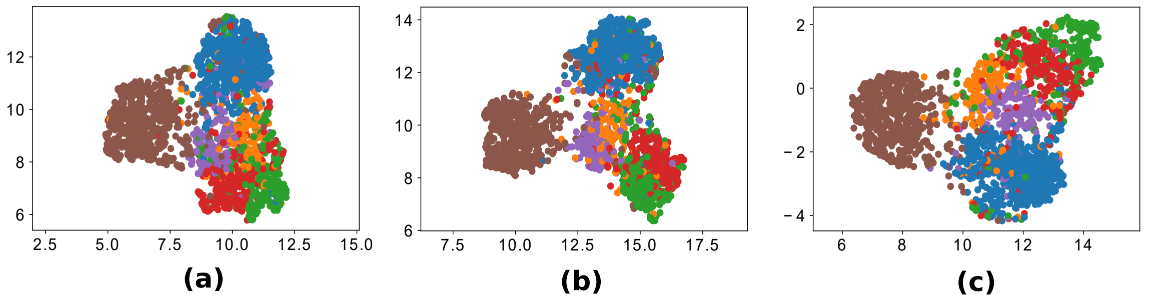

Figure S3. UMAP projection of trained latent space using our model on real WiFi CSI spectrogram data: (**a**) latent dimension=16, (**b**) latent dimension=64, and (**c**) latent dimension=128.

Table S1. Noisy measurements mean reconstruction error over a batch of 50 WiFi spectrogram data samples (full measurements considered).

Noise standard deviation	Modality 1	Modality 2
0.01	0.00347822	0.00573636
0.05	0.00348499	0.00574255
0.20	0.00349233	0.00569815
0.40	0.00354153	0.0058348
0.60	0.00357407	0.00594901
0.80	0.00367526	0.00608692

S3 Latent representation of WiFi spectrogram data

Our base model is shown in Fig. S1. The trained latent space for different latent dimensions are shown in Fig. S3. The trained latent space in Fig. S3 shows distinct clusters using UMAP (Uniform Manifold Approximation and Projection) visualization. The model was trained in a self-supervised fashion, the six clusters representing the six different human activities can be seen.

S4 Sensor fusion under noisy conditions (WiFi spectrogram data)

In this experiment, we analyze the sensor fusion performance when the data samples from the test dataset are affected by different amount of additive Gaussian noise. In this case, the measurement matrices are initialized as identity matrices with dimensions 50,176×50,176. No noise was injected to the input data from two modalities during training. The SFLR (Sensor Fusion in the Latent Representation space) algorithm (see Algorithm 1 in manuscript) is run for 1,000 iterations and the corresponding results are shown in Fig. S4, showing one sample in the test dataset. It can be observed that even under extreme noisy conditions, the noisy samples are denoised efficiently. These results are further validated in Table S1 where it can be seen that the fusion error remains essentially constant for different noise standard deviation values considering a batch of 50 images from the test dataset. Considering a batch of 50 noisy WiFi spectrograms (with additive Gaussian noise standard deviation value = 0.2), Algorithm 1 took on average approximately 22.7 seconds to converge for a given noisy WiFi CSI spectrogram sample.

S5 Sensor fusion performance with missing pixels (WiFi spectrogram data)

In this experiment, we evaluate the fusion performance of the samples under different ratios of missing pixels. The results are shown in Fig. S5 when a true data sample is randomly chosen from the test dataset and a randomly generated (binary) mask is applied to it to simulate different missing pixel ratios. Each measurement corresponds to an observed pixel. Therefore, in this case the measurement matrices will be diagonal matrices with their diagonal entries corresponding to the mask elements (1's and 0's). From Fig. S5, it can be observed that the recovered samples are very close to the true ones, even when the missing pixel ratio for both modalities is as high as 0.8. In fact, as shown in Table S2, the reconstruction error remains essentially constant with increasing missing pixel ratio for both modalities. Algorithm 1 was run for 1,000 iterations on a batch of 50 WiFi spectrograms (with missing pixel ratio = 0.4), and the optimisation process took on average approximately 9.6 seconds to converge for a given WiFi CSI spectrogram sample (with missing pixels).

Table S2. Missing pixel mean reconstruction error over a batch of 50 WiFi spectrogram data samples. Illustrations of spectrogram fusion under different missing pixel ratios are shown in Fig. S5.

Missing pixel ratio	Modality 1	Modality 2
0.1	0.00351316	0.00573596
0.2	0.00345235	0.00575154
0.4	0.00347854	0.00575382
0.6	0.003477	0.00571794
0.8	0.00349462	0.00579462

Table S3. Compressed sensing mean reconstruction error over a batch of 100 protein samples, with different noise levels.

Noise standard deviation	No. of Measurements	Modality 1 (10^{-3})	Modality 2 (10^{-3})
0.05	1 (3.125%)	52.301	55.382
	2 (6.250%)	11.678	9.200
	4 (12.500%)	0.834	0.715
	8 (25.000%)	0.387	0.450
0.1	1 (3.125%)	36.611	50.118
	2 (6.250%)	20.267	14.638
	4 (12.500%)	3.413	2.769
	8 (25.000%)	2.386	2.411
0.2	1 (3.125%)	43.466	48.271
	2 (6.250%)	17.864	19.435
	4 (12.500%)	1.528	1.435
	8 (25.000%)	5.005	5.063

S6 Toy protein dataset: additional results

Sensor fusion from subsampled and noisy toy proteins

In this section, we present the sensor fusion results for toy protein reconstruction under subsampled and noisy observations, as an extension to Section "Sensor fusion from subsampled toy proteins" in the main document. Table S3 shows the mean reconstruction error of subsampled toy protein samples, with different levels of additive Gaussian noise. The proposed SFLR method recovers both modalities from as low as 4 subsampled and noisy observations.

Sensor fusion from asymmetric compressed sensing of toy proteins

We show the results of sensor fusion from asymmetric compressed sensing, regarding the third contribution of this paper. We claim that a strong modality can be used to aid the recovery of another modality that is lossy or less informative (weak modality). Table S4 shows the recovery results in two cases. In the first case, the subsampled modality 1 with additive Gaussian noise is observed and recovered. In the second case, the noise-free modality 2 with full observation is used to help the sensor fusion. We can see that modality 2 significantly helps with the recovery of modality 1, especially when the number of observations is relatively small.

Table S4. Mean reconstruction error over 100 toy protein samples with asymmetric compressed sensing. Noise standard deviation: 0.1.

	No. of Measurements	Modality 1	Modality 2
Modality 1 with compressed sensing	1 (3.125%)	0.0542	-
	2 (6.250%)	0.0366	-
	4 (12.500%)	0.0205	-
	8 (25.000%)	0.0021	-
Modality 1 with compressed sensing<br>Modality 2 with full information	1 (3.125%)	0.0076	0.0073
	2 (6.250%)	0.0067	0.0062
	4 (12.500%)	0.0023	0.0024
	8 (25.000%)	0.0033	0.0031

References

1. Wu, M. & Goodman, N. Multimodal generative models for scalable weakly-supervised learning. In *Proceedings of the 32nd International Conference on Neural Information Processing Systems*, NIPS'18, 5580–5590 (Curran Associates Inc., Red Hook, NY, USA, 2018).
2. Xie, Y., Li, Z. & Li, M. Precise power delay profiling with commodity WiFi. In *Proceedings of the 21st Annual International Conference on Mobile Computing and Networking*, MobiCom '15, 53–64, DOI: 10.1145/2789168.2790124 (ACM, New York, NY, USA, 2015).
3. Halperin, D., Hu, W., Sheth, A. & Wetherall, D. Tool release: Gathering 802.11n traces with channel state information. *SIGCOMM Comput. Commun. Rev.* **41**, 53, DOI: 10.1145/1925861.1925870 (2011).
4. Bocus, M. J. *et al.* Translation resilient opportunistic WiFi sensing. In *2020 25th International Conference on Pattern Recognition (ICPR)*, 5627–5633, DOI: 10.1109/ICPR48806.2021.9412263 (2021).
5. Bocus, M. J. *et al.* OPERAnet, a multimodal activity recognition dataset acquired from radio frequency and vision-based sensors. *Sci. Data* **9**, 474, DOI: https://doi.org/10.1038/s41597-022-01573-2 (2022).

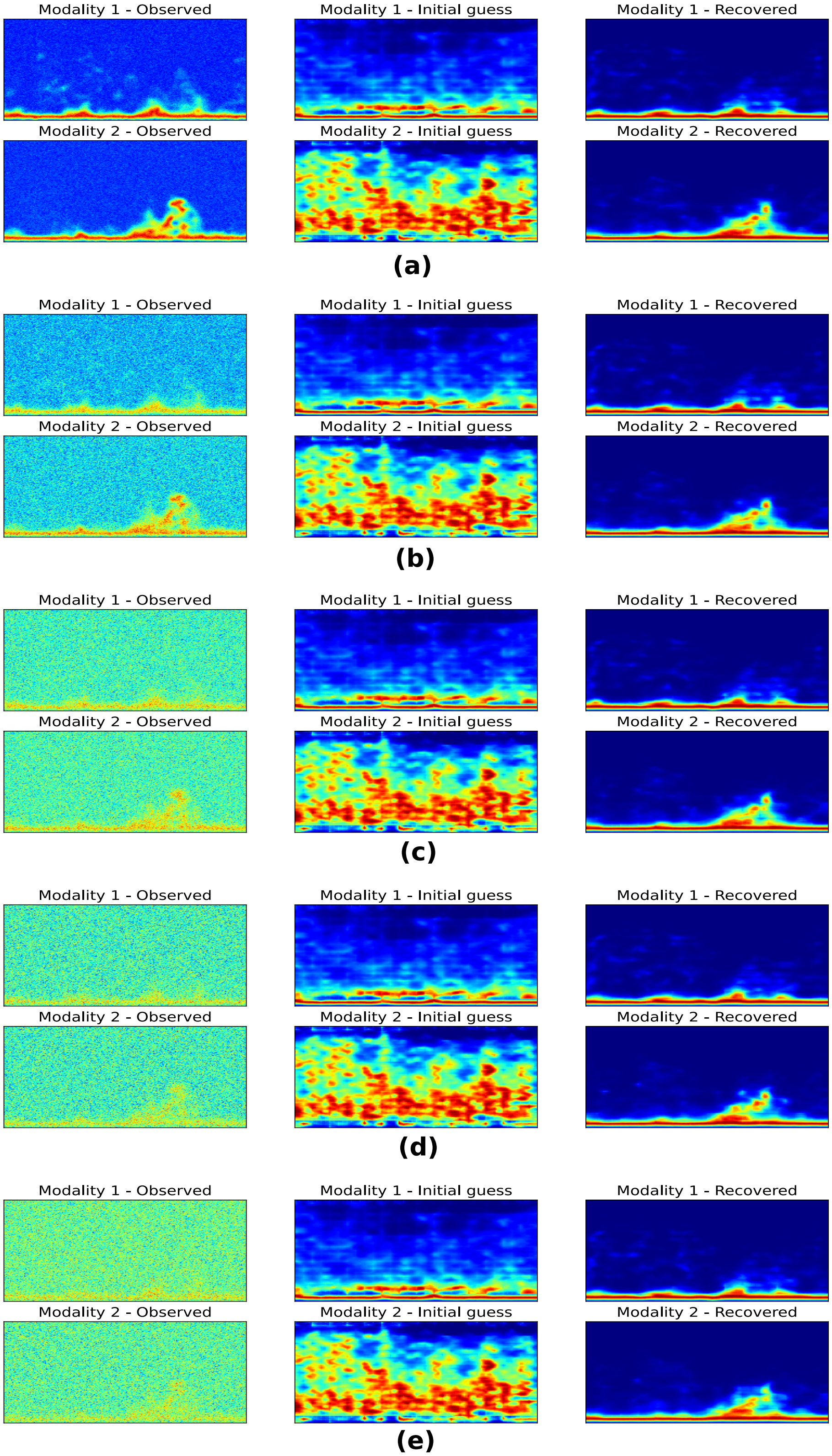

Figure S4. Impact of additive Gaussian noise on sensor fusion from the two modalities: (**a**) Std Dev=0.05, (**b**) Std Dev=0.2, (**c**) Std Dev=0.4, (**d**) Std Dev=0.6, and (**e**) Std Dev=0.8. Left column shows noisy spectrogram sample, middle column shows fusion with initial guess (no optimization) while right column shows fusion with $\hat{z}_{MAP}$.

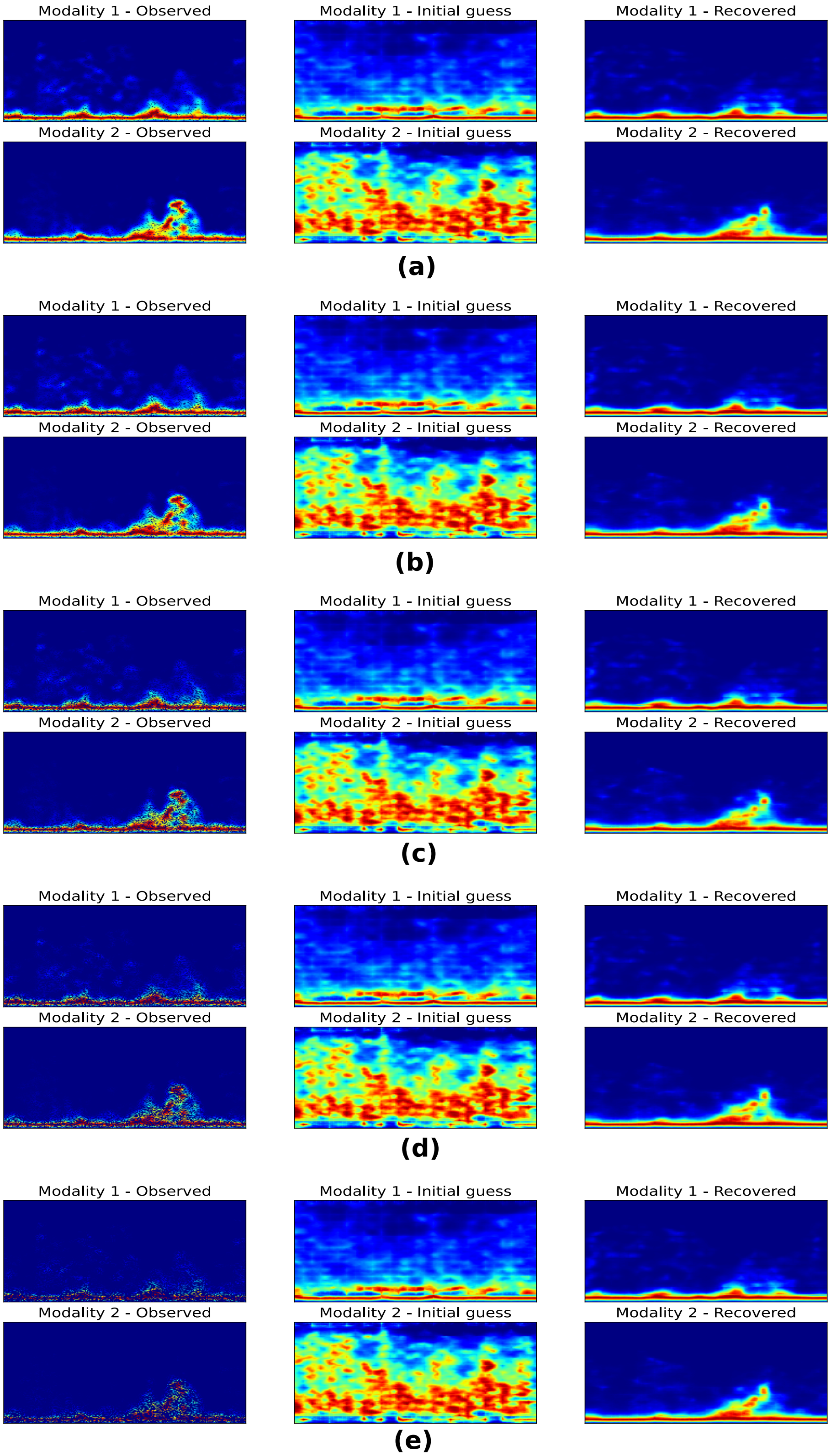

Figure S5. Impact of missing pixels on spectrogram recovery from the two modalities (no additive Gaussian noise): (**a**) missing pixel ratio=0.1, (**b**) missing pixel ratio=0.2, (**c**) missing pixel ratio=0.4, (**d**) missing pixel ratio=0.6, and (**e**) missing pixel ratio=0.8. Left column shows spectrogram sample with missing pixels, middle column shows reconstruction with initial guess (no optimization) while right column shows reconstruction with $\hat{z}_{MAP}$. Very good recovery performance is observed in all cases.

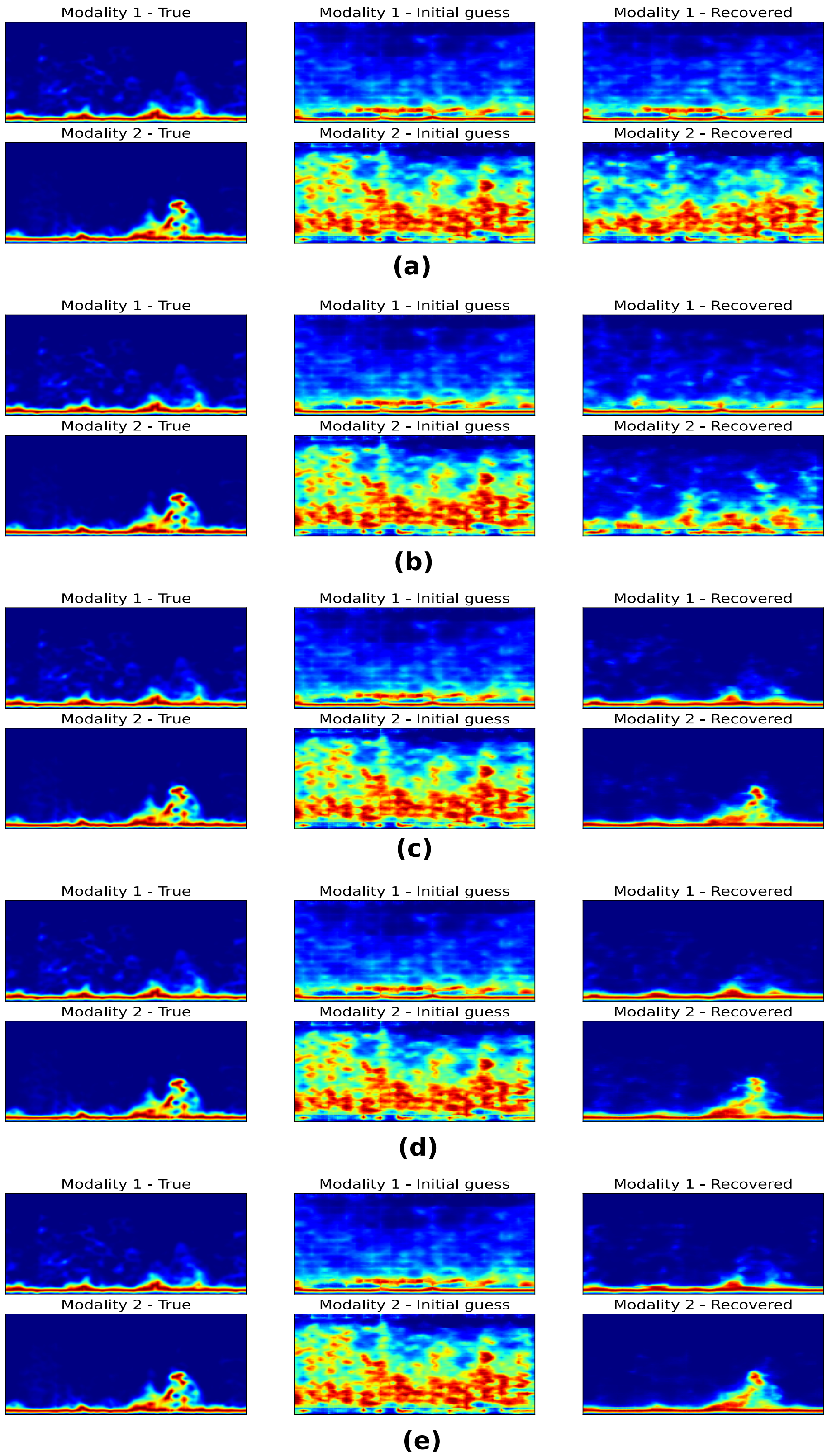

Figure S6. Compressed sensing performance on different number of measurements without additive Gaussian noise: (**a**) 1 measurement out of 50,176 (0.002%), (**b**) 10 measurements out of 50,176 (0.02%), (**c**) 196 measurements out of 50,176 (0.39%), (**d**) 784 measurements out of 50,176 (1.56%), and (**e**) 1,568 measurements out of 50,176 (3.125%). Left column shows true spectrogram sample, middle column shows reconstruction with initial guess (no optimization) while right column shows reconstruction with $\hat{z}_{MAP}$.
